# Towards the integration of digital cytology in the tablet technologies

**DOI:** 10.1186/1746-1596-8-S1-S40

**Published:** 2013-09-30

**Authors:** Daniele Giansanti, Marco Pochini, Enrico Giarnieri, Maria Rosaria Giovagnoli

**Affiliations:** 1 Istituto Superiore di Sanità, Roma, Italy; 2 Università Sapienza, Facoltà di Medicina e Psicologia, Roma, Italy

## Background

The today’ s technologies allow an easy way to share information relevant to image by means of different tools. Many solutions are today available that allow to the cytologist to exchange digitalized information about glasses. It is thus arising the need of objective methodologies, such as the ones based on HTA that we have proposed in a previous study focused in tele-pathology [1], to investigate the potentialities of the new technologies in D-CYT. It should be also considered that the D-CYT will have a great impact in the work organization as the interaction with the glasses is radically changing [2] giving also new chances to e-learning [3-5].

In a typical today’s architecture there is

a) an *on-site-server* with the scanner or alternatively a tele-pathology *third-party-server* (in site or in remote as a Web service such as the Leeds’ centre [6] at http://www.virtualpathology.leeds.ac.uk/index.php), for the creation of the *virtual glasses* named Digital Slides (DS)s,

and

b) low cost (or free) *Light-client applications* proprietary software tools (PC-client application not asking enough hard disk space, not consuming dynamically PC resources, such as RAM, SWAP ecc., simple and user-friendly) for the navigation on the DS which can be installed in remote clients.

This new methodology is rapidly largely spreading and it is becoming the core aspect of the formation for future qualified personnel and is more and more asking for user-friendly and effective ICT solutions.

The *Tablet-technology-ICT-solution* is recently widely increased as a user-friendly and effective tool to remotely share image information. Thanks to this technology it is possible to navigate into an image (pan, zoom-in, zoom-out) using only the hand-fingers without typing the keyboard or the software interfaces’ keys. This way of image navigation is going to further improve the application of Telemedicine to DP and in particular in D-CYT with particular reference to the remote and/or cooperative decision and diagnosis in cytology.

## Material and methods

The methodological flow clearly arises by inviting the reader to navigate in the Public WEB of the University of Leeds named “Virtual Pathology at the University of Leeds” where several digital-slides are available for public use at the URL :

http://www.virtualpathology.leeds.ac.uk/public/common_slides.php

The reader can try to navigate the DS by means of a PC using the interface of Spectrum Web Viewer (by Aperio) or by means of a Tablet system using the fingers and *subjectively* consider thus the differences. It is thus clear that there is thus strongly the need of considering both the state of art of the Tablet technologies and the design of an *objective methodology* to assess the technology. The methodological flow faced thus the two basic issues:

• Analysis of the state of art of the tablet technologies.

• Investigation of HTA solutions [1] to assess the technology (Tablet and applications of D-CYT) both in terms of performances and acceptance for the relevant introduction in Telemedicine [6-8], [9,10].

## Results and discussion

### Analysis of the state of art of the tablet technologies

The analysis with the focus to D-CYT returned that the tablet technologies could be grouped into: *wearable* tablets, *portable* tablets and *not-portable* tablets.

**Figure 1:**
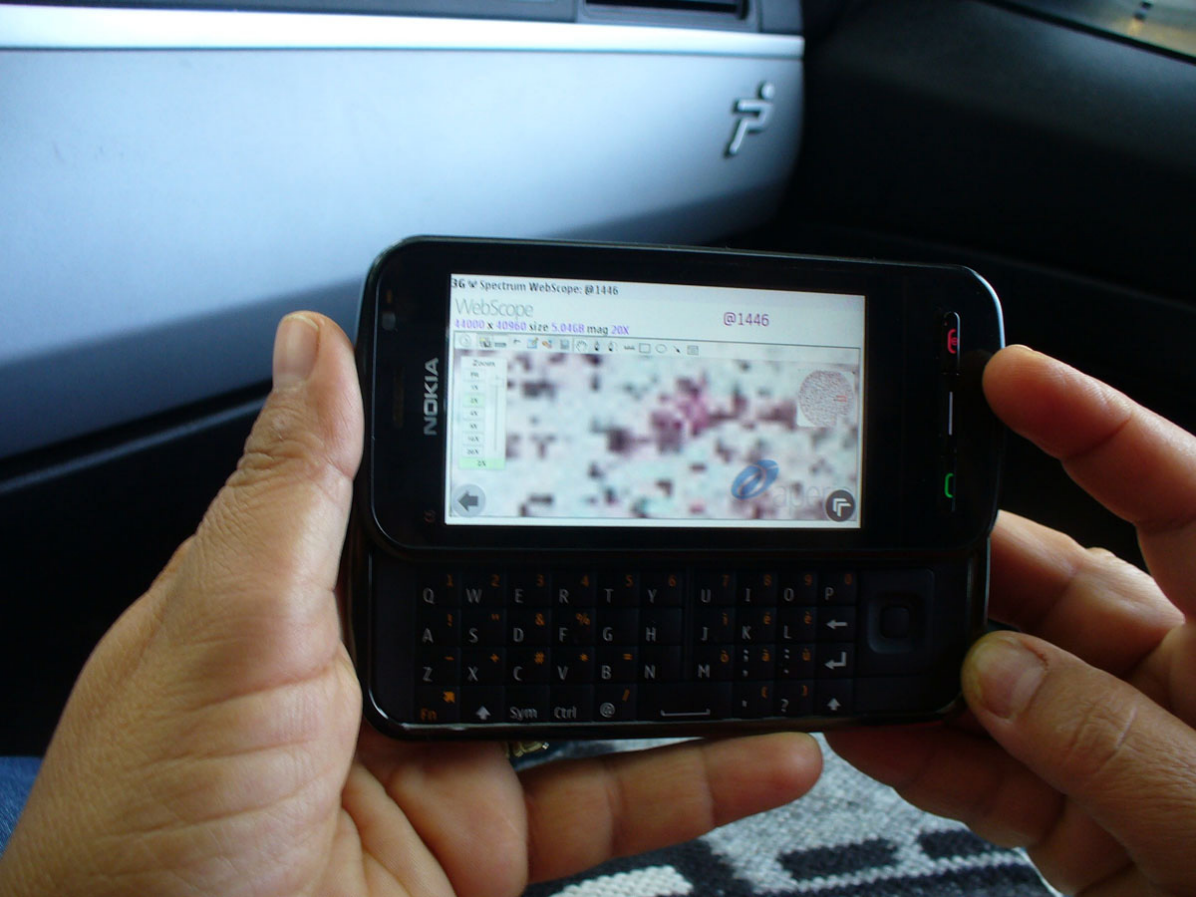
Navigation using the WEB-Scope by Aperio using a wearable tablet

**Figure 2:**
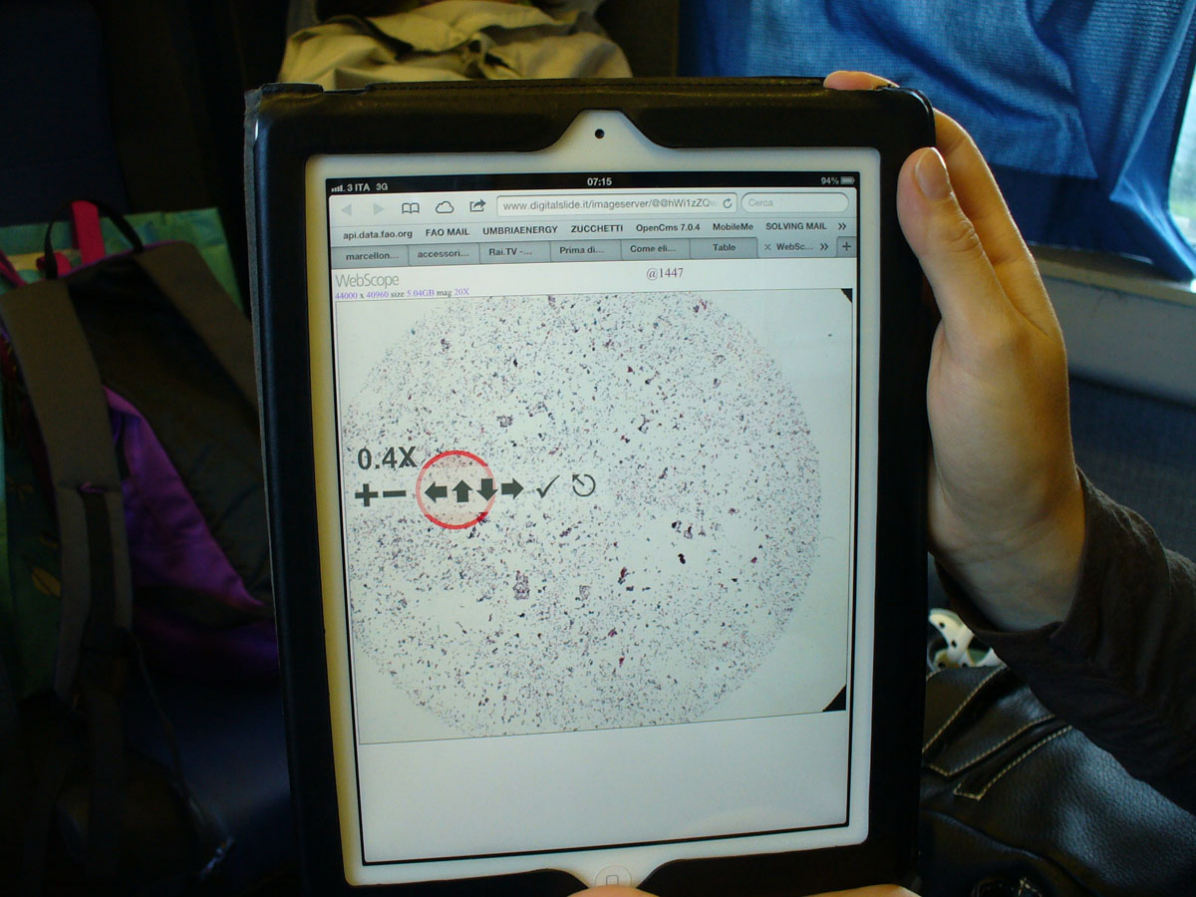
Navigation using the WEB-Scope by Aperio using a portable tablet

- The *wearable* tablets comprehend the Smart-phones i.e the devices that can be embedded in a pocket (Figure 1 ).

- The *portable* tablets comprehend the *A4 A4/2* tablets such as the Apple Ipad i.e the devices that can be embedded in a 24-hours-suitcase (Figure 2).

- The *not-portable* tablets comprehend the very large touch tablets such as the XDesk or Microsoft Surfaces, i.e the devices that cannot be self carried.

The first two systems, that are widely used for many different purposes allow to reach everyone in the world, therefore represent a chance for the remote consulting in D-CYT (Figure 1,2).

The last systems, focusing for example on the Epson XDesk, represent a powerful ICT solution for cooperative analysis and discussion of cases of virtual cytology.

**Figure 3:**
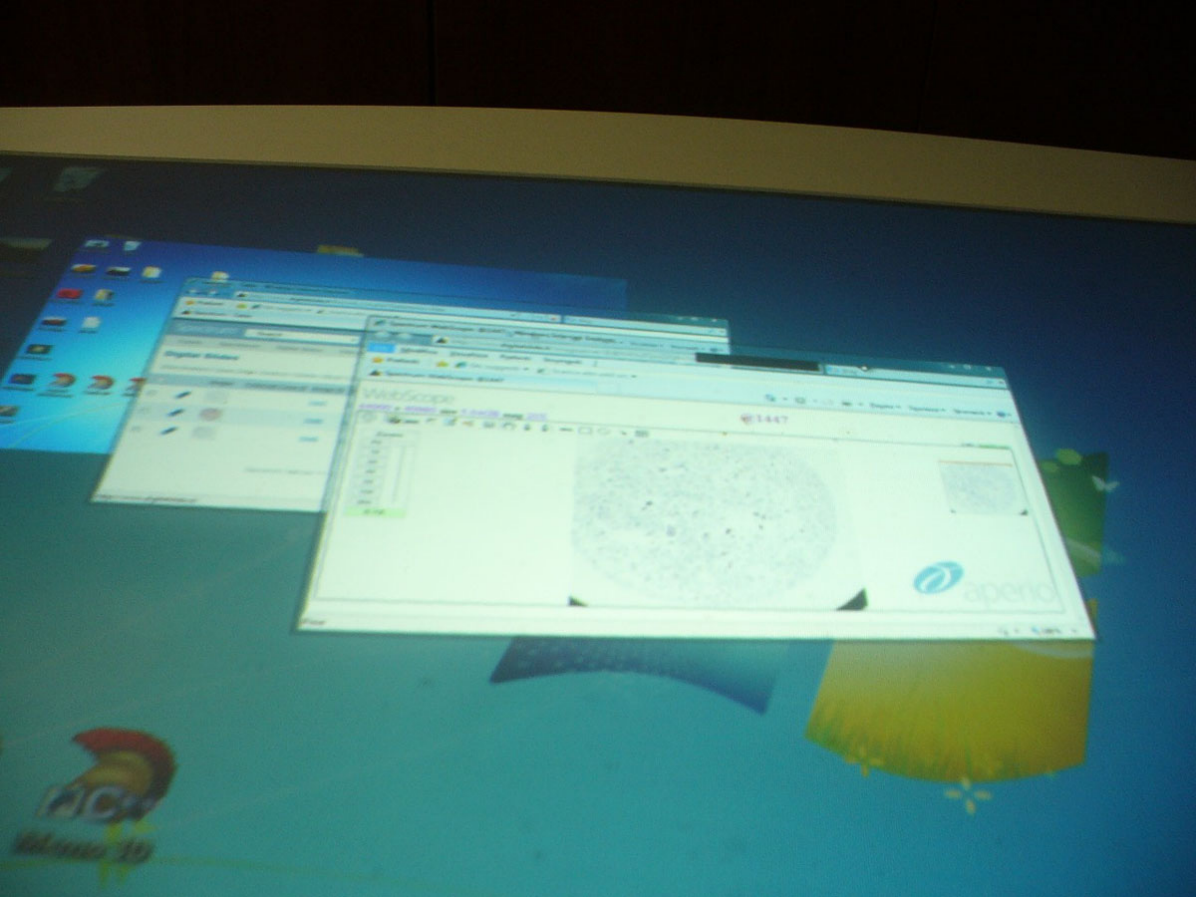
Cooperative scenario in D-CYT with the Xdesk : The Windows

**Figure 4:**
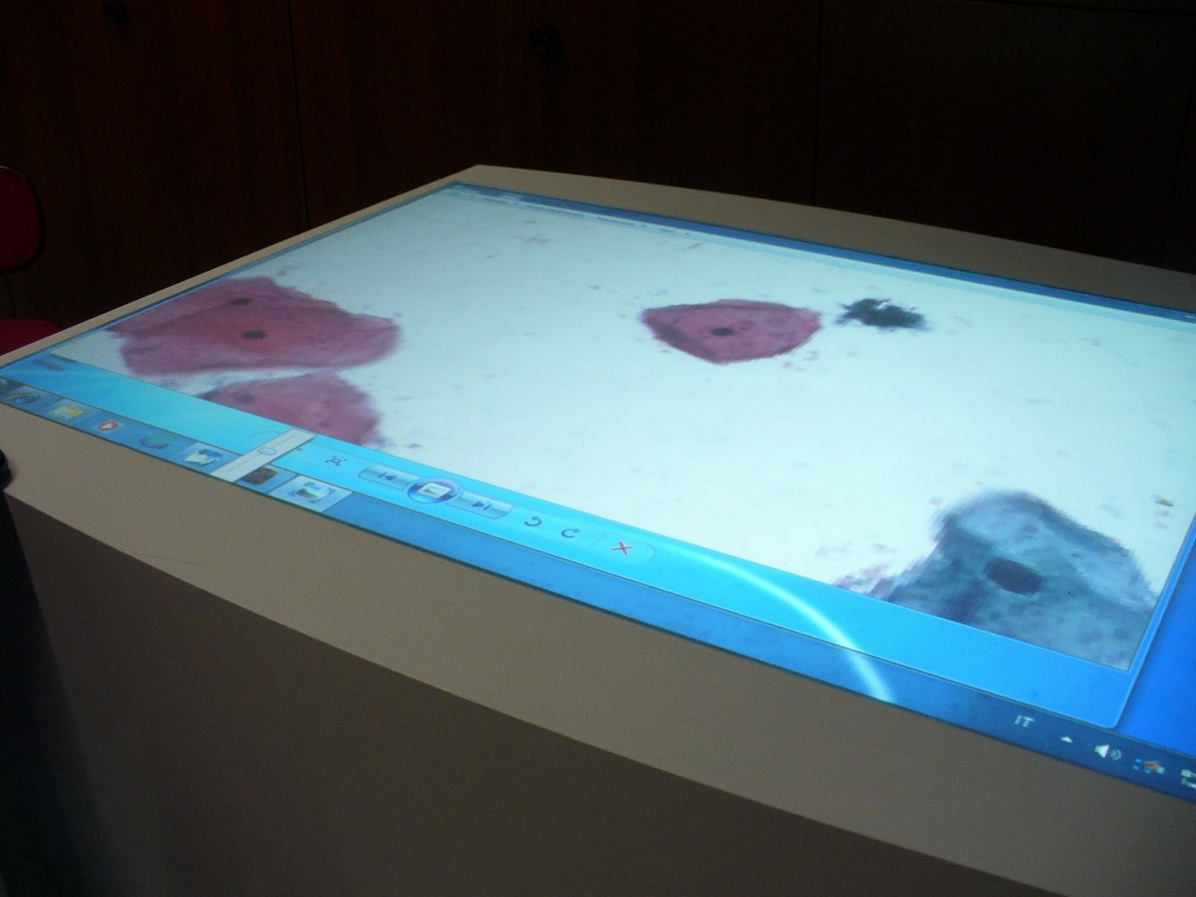
Cooperative scenario in D-CYT with the Xdesk : The virtual-slide

**Figure 5:**
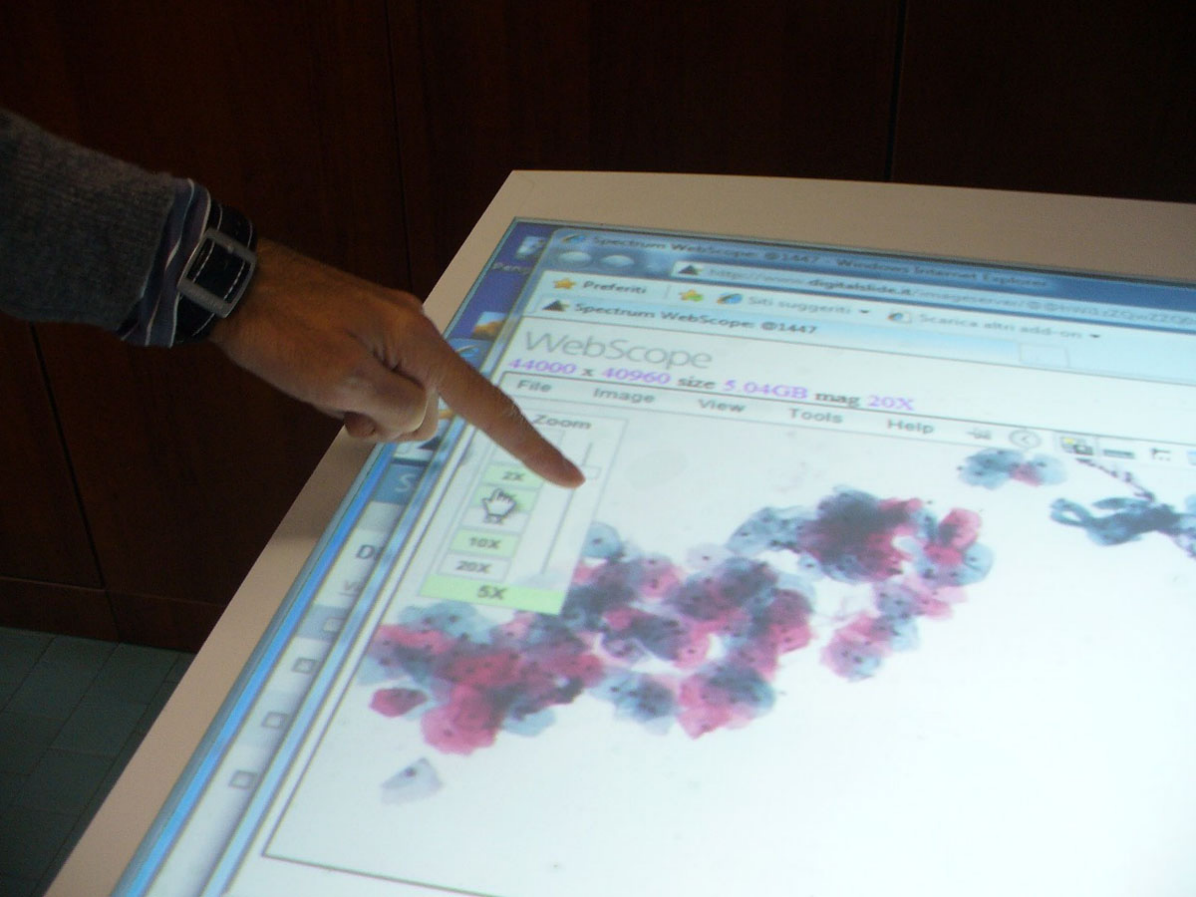
Cooperative scenario in D-CYT with the Xdesk : The navigation based on the fingers

In details, the Epson XDesk is an interactive table; some call it a coffee table because you can put anything on the surface of the table, it works by projections, with the very latest technologies on that. This desk is also compatible with Bluetooth communication protocol and as soon as you put your phone or camera on the surface of the table, the XDesk will be able to see all your files and pictures on the desk. By natural interface pictures on the table can be managed freely and resized, zoomed in and out by finger movements as the iPod touch does, only on a fair larger scale. The Epson XDesk has a 52-inch screen and a 1024x768 touch screen display. It represents the appropriate high technology solution for cooperative discussions (Figure 3,4,5), clinical audit and ultimately the future direction of cooperative virtual microscopy environment. Furthermore it could represent a tool suitable to recover the inheritance of some ICT solutions (such as Pap-Net) for large screening in cytology abandoned because in the first applications the technology was not ready, for computer assisted cervical/vaginal cytology diagnosis [7].

### Investigation of HTA tools to assess the technology

As a core aspect is the introduction of the new technologies for the D-CYT in the Hospital, specific studies of Health technology assessment should be performed focused in the new technologies for the digital-cytology. This is a basic aspect for the Health Care Systems.

**Figure 6:**
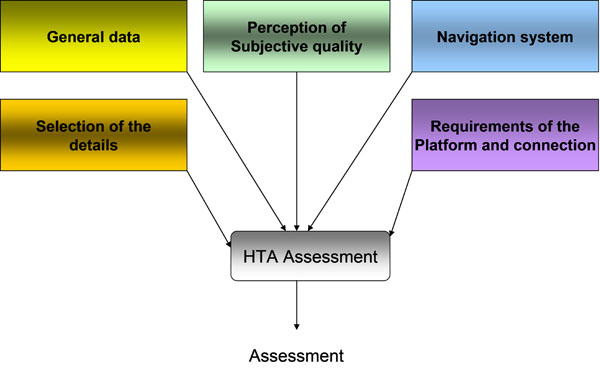
Key points investigated by means of the HTA tool

As a HTA tool we recovered the interactive word based tool we have proposed in [1] for applications in tele-pathology. This tool had been proposed before the diffusion of the tablet technologies and was thus conceived for the assessment of *PC-based-technologies* for D-CYT. However the tool is portable, as it is, on *tablet based technologies*. Figure 6 elucidates the contents of the HTA tool, each element contains a number of questions with a scoring based on 4 levels. Starting from this tool the investigation will be widened considering also:

1. Further issues relevant to studies of HTA, as from specific experience of mondial networks of HTA such as the EUNETHTA (http://www.eunethta.net/) .

2. Integration of specific studies on HTA over the NET, as the focus is the D-CYT is the communication over the WAN/LAN.

## Conclusions

Tablet technologies have been reviewed with the focus to the perspectives of the D-CYT. A HTA specific tool has been proposed to assess the performances and acceptance of the applications in D-CYT based on tablet technologies. Possibilities and limitations of the three different tablet technologies will be deeply investigated by means of the proposed HTA methodology on experts and students approaching the new scenario of digital-cytology.

## List of abbreviations used

HTA: Health Technology Assessment; DP: Digital Pathology; D-CYT: Digital Cytology; DS: Digital Slide

## Competing interests

The authors declare that they have no competing interests.

## Authors’ contributions

DG MRG wrote the manuscript as major contributors. MP has prepared the review on the wearable and portable and not portable Tablet technologies and is currently caring the experimentation study. EG has reviewed and improved the scientific content and rationale of the manuscript. All authors have read and approved the final manuscript.
